# Barriers and enablers to implementing ‘DEALTS2’ simulation-based train-the–trainer dementia training programme in hospital settings across England: a qualitative study

**DOI:** 10.1186/s12913-021-06977-1

**Published:** 2021-09-09

**Authors:** Michelle Heward, Michele Board, Ashley Spriggs, Dina Blagden, Jane Murphy

**Affiliations:** 1 Ageing and Dementia Research Centre, Faculty of Health & Social Sciences, Bournemouth University, 10 St Pauls Lane, BH8 8GP Bournemouth, UK; 2 Nursing for Long-Term Health Research Centre, Faculty of Health & Social Sciences, Bournemouth University, 10 St Pauls Lane, BH8 8GP Bournemouth, UK

**Keywords:** Dementia, Training, Simulation, Train-the-trainer, Acute care, Implementation, Qualitative, Clinical practice, Barriers and enablers, Knowledge and confidence

## Abstract

**Background:**

Despite approaches to provide effective dementia training in acute care settings, little is known about the barriers and enablers to implement and embed learning into practice. We were commissioned by Health Education England to develop and evaluate a new dementia training intervention ‘Dementia Education And Learning Through Simulation 2’ (DEALTS2), an innovative simulation toolkit to support delivery of dementia training in acute care across England. This study aimed to explore barriers and enablers experienced by trainers implementing DEALTS2 and extent to which it impacted on delivery of training and staff clinical practice.

**Methods:**

We conducted twelve one-day DEALTS2 train-the-trainer (TTT) workshops across England in 2017 for National Health Service Trust staff employed in dementia training roles (n = 199 trainers); each receiving a simulation toolkit. Qualitative data were collected through telephone interviews 6–8 months after TTT workshops with 17 of the trainers. Open ended questions informed by the Kirkpatrick model enabled exploration of implementation barriers, enablers, and impact on practice.

**Results:**

Thematic analysis revealed six themes: four identified interrelated factors that influenced implementation of DEALTS2; and two outlined trainers perceived impact on training delivery and staff clinical practice, respectively: (i) flexible simulation and implementation approach (ii) management support and adequate resources (iii) time to deliver training effectively (iv) trainer personal confidence and motivation (v) trainers enriched dementia teaching practice (vi) staff perceived to have enhanced approach to dementia care. Trainers valued the DEALTS2 TTT workshops and adaptability of the simulation toolkit. Those supported by management with adequate resources and time to deliver effective dementia training, were likely to implement DEALTS2. Trainers described positive impacts on their teaching practice; and perceived staff had enhanced their approach to caring for people with dementia.

**Conclusions:**

Trainers explained individual and organisational barriers and enablers during implementation of DEALTS2. The flexible simulation and implementation approach were key to supporting adherence of DEALTS2. To ensure wider implementation of DEALTS2 nationally, Trusts need to allocate appropriate time to deliver effective dementia training. Future research should measure staff behaviour change, patient perspectives of the intervention, and whether and how DEALTS2 has improved health and care outcomes.

## Background

A quarter of hospital beds in England are occupied by people with dementia [1]. Yet staff do not always feel that confident in their ability to effectively support patients with dementia [2]. This can have a negative impact on the quality of care and support provided to people with dementia and their families in acute care settings. Staff need to understand how they can enhance the care they deliver to people with dementia and ensure that it is person-centred [3–7]. Providing effective dementia training is one way that hospitals can ensure their staff have the appropriate knowledge and skills to deliver good person-centred dementia care [1, 8–11]. However, dementia training for hospital staff is not mandatory in the UK, despite policy documents outlining its importance [6]. This has led to differences in the approaches used to deliver dementia training (ranging from workshops/study days to eLearning, workbooks, and higher education modules) which has affected the quality of the training being delivered [6, 12]. Moreover, indicating a wider need to improve the quality and consistency of dementia training in UK hospitals.

### Innovative simulation training to ‘put staff into the shoes of a person with dementia’

In healthcare fields the use of simulation training has long provided a safe space for staff to practice real-world situations with an opportunity to reflect on behaviours and responses and ensure that this supports the development of their practice [13, 14]. Simulation training allows staff to feel more confident when they go on to face similar situations in their own practice and is a safer option for patients as it evades unnecessary risks [13, 14]. Some scholars have noted that simulation training can enable staff to become emotive about the topic which is useful for enhancing person-centred care [15–18]. Simulation has been deemed a valuable training approach for staff in hospitals to ‘put themselves into the shoes of a person with dementia’ [15, 17]. Although, to date there appears to be a lack of simulation training programmes focused on the topic of dementia for use in hospital settings [19]. More research is therefore needed to identify what leads to effective implementation of dementia focused simulation training, and why and how such training makes a difference to the staff being trained and the care they deliver [2, 20].

### Study rationale

In direct response, we were commissioned by Health Education England (HEE) to create a new national innovative dementia training intervention, in the form of a simulation toolkit, to support trainers to utilise innovative dementia training approaches across England. Building on a pilot version ‘Dementia Education And Learning Through Simulation’ (DEALTS) programme created by HEE [21, 22], we developed ‘Dementia Education And Learning Through Simulation 2’ (DEALTS2) using an approach that enabled us to apply learning from previous studies and allowed feedback to inform the content development [23]. Given the national scale of the study, understanding trainer experiences of barriers and enablers to implementing the DEALTS2 innovative dementia training approaches is of value to those involved internationally in dementia training, and healthcare workforce development more broadly.

### DEALTS2 intervention and implementation

DEALTS2 is a simulation-based dementia training intervention, placing staff into the shoes of a person with dementia to facilitate positive impacts on practice. DEALTS2 uses Kolb’s [24] four stage experiential learning cycle to frame the process of learning. This involves placing all hospital staff into the shoes of a person with dementia to gain an insight into the lived experience and reflecting on that experience to support the development of interpersonal skills with the aim of facilitating positive impacts on practice [15, 17]. Mapped to Tier 2 of the Dementia Training Standards Framework (DSTF) [25, 26] covering three subjects, risk reduction and prevention, person-centred care, and communication. DEALTS2 is designed to support the delivery of dementia training beyond general awareness level (Tier 1) for staff with regular contact with people with dementia (including Nurses, Medical Practitioners, Allied Health Professionals, Health Care Assistants, Porters and Receptionists). Theoretically the training is underpinned by the Humanising Values Framework (HVF), a philosophical lens that identifies potentially humanising and dehumanising elements in caring systems and interactions [27]. Integrating the HVF within DEALTS2 allows staff to reconsider what it means to be human, and ultimately reflect this learning in their approach to patients, colleagues, and the value of their own contribution [28].

The DEALTS2 dementia training intervention was delivered across England in 2017 through a Train-The-Trainer (TTT) model with trainers (n = 199) participating [23, 29]. Trainers were responsible for implementing DEALTS2 into the dementia training in their employing NHS Trust and the materials were adaptable to suit local learning needs. To support this, trainers received the DEALTS2 simulation toolkit [30] which was reviewed as part of the pilot of the Dementia Training Design and Delivery Audit Tool (DeTDAT) [31], to ensure it met good practice criteria identified in the ‘What Works’ in dementia training study [2, 32].

S*tudy aim and objectives*.

While literature outlines key components of effective dementia training in acute care settings [2, 6, 7, 18, 20, 32]; a paucity of studies examine the barriers and enablers to implementing learning in practice and the persistence of practice change beyond the immediate timeframe of the training [20]. The aim of the overall study was to evaluate the implementation and impact on practice of the ‘DEALTS2’ dementia training intervention in acute care settings across England. The research objectives were to:

1. Evaluate differences in dementia knowledge scores pre and post training, satisfaction with DEALTS2 train-the-trainer workshops and simulation toolkit, confidence to use the innovative training approaches, and spread of implementation.
2. Examine enablers and barriers experienced by trainers to implementing DEALTS2 across England to identify factors that determine successful implementation and the resulting changes to practice.

## Methods

### Study design

This study was conducted in two phases [23]. Quantitative data from the questionnaire survey in response to Objective 1 is reported elsewhere [29]. In this paper we report on qualitative data collected in response to Objective 2 through semi-structured telephone interviews that took place six to eight months after the DEALTS2 TTT workshops. This period was selected to give trainers time to reflect on their learning from the TTT workshops and have an opportunity to use the DEALTS2 materials in their dementia training. This therefore enabled trainers to feedback in the interviews on barriers and enablers they had experienced.

### Participant recruitment

We aimed to conduct telephone interviews with at least one trainer from each of the 12 HEE regions participating in the TTT workshops, to ensure representation from across England. Using a simple random sample [33], potential participants were assigned an identification number and selected using an online random number generator, from those who gave their consent to be contacted during the DEALTS2 TTT workshops. Initial contact was made by telephone and those who agreed to take part booked a suitable interview date and time with the researcher and were then emailed information about the study. Three attempts were made to contact participants by telephone, after which contact with the next potential participant was then established. This process was repeated until the point where all those who had given consent had been contacted, or data saturation, if that was sooner.

We invited 29 of the trainers that attended the DEALTS TTT workshops in 2017 (*n* = 199) to take part in a telephone interview. In the end, 17 interviews were completed with representation from 11 of the 12 HEE regions (Table 1). Most of the trainers were employed in acute care 41 % (*n* = 7), and the other trainers in community hospitals 29 % (n = 5), social care 24 % (n = 4), and voluntary sector 6 % (*n* = 1). Just over half of the sample, 59 % (*n* = 10), were already using materials from the DEALTS2 toolkit in the dementia training they were delivering in their Trust, whilst the remaining 41 % (n = 7) were not yet using them.

**Table.1:** Number of trainers taking part in interviews, by Health Education England region

Health Education England regions during DEALTS2 workshop delivery in 2017 (*n* = 12)	Number of participants per Health Education England region	
	**Attended train-the –trainer workshops (*n* = 199)**	**Participated in a telephone interview (*n* = 17)**
East of England	24	2
North West London	19	1
South London	28	2
North Central and East London	18	0^a^
East Midlands	18	1
West Midlands	18	2
North East	13	1
Yorks and Humber	18	1
North West	21	1
Thames Valley	0^b^	0^b^
Kent, Surrey and Sussex	7	3
South West	13	2
Wessex	17	1
Total	199	17

^a^unable to recruit from this region

^b^did not try to recruit from this region as they took part in pilot

### Method

Data collection and analysis was informed by the Kirkpatrick model [34] for evaluating effectiveness of training [23]. Telephone interviews were conducted by one of the authors (DB) and ranged from 15 to 32 min, excluding the consent processes. Whether or not the trainers were already using the DEALTS2 toolkit, together with the amount of time they were able to set aside for the interview during their working day, impacted on the length of time the interviews took to complete. Qualitative questions were designed to explore the barriers and enablers experienced by trainers to implementing DEALTS2 in their own dementia training, and for those who had implemented DEALTS2 the perceived impact on their own practice as educators, staff clinical practice, patient care and wider service improvement (Table 2).

**Table.2:** Interview Guide

Discussion topic	Questions	Evaluation type (Kirkpatrick, 1959)
Adaption and/or adoption of DEALTS2 materials in own dementia training	• Which elements of DEALTS2 are you using in your own dementia training and how are you adapting the materials to suit your own/organisation needs? • How are staff responding to the DEALTS 2 simulations in your training sessions? • How are you finding delivering the debriefing sessions after the simulations?	Behaviour
Facilitators and barriers to implementing DEALTS2 materials in own dementia training	• What has helped you to implement the DEALTS 2 toolkit in your organisation? • What have you found difficult about implementing the DEALTS 2 toolkit in your organisation? • What might help you to implement the DEALTS 2 toolkit in your organisation? • Is there any further support or information that would help you to implement the DEALTS 2 toolkit in your organisation?	
Perceived impact of DEALTS2 on delivery of training and staff clinical practice	• What changes have you noticed, or been informed of by others, following the introduction of the DEALTS 2 materials in the dementia training delivered in your employing Trust? *(Probe for examples to illustrate the answer)* a. Impact on your own performance/ practice as educators b. Perceived impact on staff clinical practice c. Perceived impact on patient care d. Impact on service improvement	Results

### Ethics and consent procedure

 Ethical approval was obtained from Bournemouth University Research Ethics Committee (Reference ID 17,647). All methods were carried out in accordance with local guidelines and regulations [35]. Local ethics and governance processes were followed, including principles of informed consent, voluntary participation, the right to withdraw, confidentiality and anonymity ensuring data is stored securely and retained or destroyed in accordance with the Data Protection Act 1998, were adhered to. Trainers participating in telephone interviews were emailed a participant information sheet and asked for their written or verbal agreement to participate prior to the start of the interview. Responses to telephone interview questions were recorded in writing by the interviewer (DB) in Microsoft Excel (version 2002) during the interviews and were expanded in full immediately after each interview. All identifiers were later removed ready for analysis.

### Data analysis

Data was analysed using an inductive six phase thematic approach, as outlined by Braun and Clarke [36]. The analysis was inductive, in that themes emerged from the data as the analysis progressed, rather than imposing a theoretical framework to the data. However, we did deductively impose the following overall framework to ensure the analysis directly addressed the study research objectives: (1) implementation barriers and enablers, and (2) impact on training delivery and staff clinical practice. The initial stages of analysis were conducted by two researchers (MH and DB) and the emerging themes were then reviewed and discussed by the whole research team (all authors) until a consensus was reached. Detailed discussions were undertaken throughout this process to provide rigour to the analysis. Themes that reflected the research questions and were frequently cited were considered significant [37]. Analysis was written up using key quotations to support the themes in the trainer’s own words.

### Findings

Six themes were constructed from the data (Table 3). The first four themes identify the interrelated factors that influenced whether trainers were able to implement DEALTS2 into practice. The remaining two themes discuss impact on training delivery and staff clinical practice, identified by trainers who had implemented DEALTS2. Each theme is discussed in turn.

**Table.3:** Overview of themes

Overall analysis framework	Themes
Implementation barriers and enablers	1. Flexible simulation and implementation approach
	2. Management support and adequate resources
	3. Time to deliver training effectively
	4. Trainer confidence and motivation
Impact on practice	5. Trainers enriched dementia teaching practice
	6. Staff perceived to enhance dementia care approach

### Implementation barriers and enablers

Trainers were asked to discuss what factors had helped or hindered them from being able to implement DEALTS2 in their own practice. The factors described by trainers were related to the individual and the organisational level.

#### Theme 1 - Flexible simulation and implementation approach

Trainers suggested that the flexibility of the DEALTS2 implementation approach was a key aspect that facilitated use in practice. Trainers valued the opportunity to meet colleagues from other Trusts in their region to share the learning experience, obtain the DEALTS2 simulation toolkit and facilitate future networking:

*‘Attending the DEALTS2 training and getting the slides/resources [was useful].’ (Interview 7)*

*‘[I have] appreciated being ‘given time to attend the workshop’ and ‘prepare for the training sessions’ (Interview 5).*

Regardless of their prior experience of delivering dementia training, trainers found the DEALTS2 TTT workshops informative and useful for providing them with new ideas for delivering dementia training. For some trainers the content was novel and insightful; for others it helped to fill in the gaps, or validated alignment with current in house dementia training programs which they felt was reassuring for them to know that what they were already delivering was aligned to current evidence and best practice:

‘… *it has really enriched me as a trainer, there are some very good resources… which will be used. (Interview 14).*

*‘I am already delivering similar training… [DEALTS2 resources] are all very similar to what I am already using’ (Interview 15).*

Trainers appreciated the adaptability of the simulation toolkit which could be tailored to individual Trust training needs. The ability to adopt the toolkit as is, or to adapt it to suit, meant that it was useful to a larger number of trainers. It enabled those with different level of previous experience of delivering dementia training to be able to apply the innovative dementia training approaches to their own practice. Trainers reported slight variance in their use of the simulations from how they were originally set out in the DEALTS2 simulation toolkit, this involved amending the description of the role play for example to suit the staff they were training. The DEALTS2 toolkit supported this, enabling them to identify what they thought would work best with a specific group of staff:

*The role play simulations… got good feedback for this… good scenarios in relation to communications (Interview 5).*

*All of them are relevant and they all have some good bits… I will use most of them…, however have to amend/rewrite some of them (Interview 3).*

*‘I have adapted them all and I am looking at adding more from the [toolkit]. We have found [some simulations have] not been suitable for everyone……’* (Interview participant 16).

In contrast, other trainers appeared to find transferability of the materials more difficult, particularly if the examples were not directly in an acute care setting, or if they did not know which members of staff would be attending the training ahead of the session:

‘…*the [care home] video… would have worked in a different setting… it was difficult to adapt and fit it to all staff’* (Interview 5).

‘*As I did not have the full background of the staff who were attending the training, I was unsure of what they were expecting to get from this training’* (Interview 7).

Moreover, some trainers wanted DEALTS2 to cover more subjects from the DSTF to support deliver of the whole of tier 2:

*‘[DEALTS2] only covers 3 [Dementia Standards Training Framework] areas… ideally the sessions should cover all Tier 2 subjects’. (Interview 1)*

Collectively though, trainers felt that the versatility of the simulations and implementation approach meant that they could adapt DEALTS2 to suit the training needs in their Trust, and this increased the likelihood that they would use it in practice.

#### Theme 2 - Management support and adequate resources

Trainers described how feeling empowered by management to use the DEALTS2 simulations and supported with adequate resources (such as equipment, physical space, and colleagues to help deliver dementia training sessions) was fundamental to them actually using DEALTS2 in their dementia training. Many trainers described how after attending the DEALTS2 TTT workshops they had to seek approval from their line manager before being able to use the DEALTS2 simulation toolkit in their dementia training. However, for others they reported seeking approval was more difficult, particularly if line managers or senior management did not see the value of simulation training in dementia care:

‘…a*s soon as we got the all clear from higher up [senior managers] we were able to start delivering the 4-hour sessions.’ (Interview participant 15).*

*‘I had to wait for management to say that we could go ahead with the training’ (Interview 13).*

Being assigned responsibly to implement DEALTS2 in their Trust, and appropriate resources (equipment, physical space, and colleagues to help deliver dementia training) to enable this, was pivotal in trainer use of DEALTS2 in practice.

*… ‘that I have been in charge of implementing the training. That I have the equipment and staff that is needed to provide with training’. (Interview 9)*

*‘…having sufficient staff to be able to deliver the training [has helped us implement DEALTS2]’. (Interview 16)*

Conversely, in Trusts where trainers suggested that the above resources were lacking, trainers were less likely to report using the DEALTS2 materials. Limited staff to deliver dementia training in one Trust meant they were moving away from face-to-face training delivery towards e-learning and so using the DEALTS2 simulation toolkit based on face to face delivery methods was not an option for them.

#### Theme 3 - Time to deliver training effectively

Trainers described how the length of time they were allocated by their Trust to deliver higher level dementia training was less than recommended to adopt the full DEALTS2 training package (approximately four hours for the three subjects). In some Trusts trainers reported having an hour and a half to delivery Tier 2 dementia training. These Trusts appeared to undervalue the time it takes to deliver effective dementia training beyond general awareness level (Tier 1). The consequence of this was that trainers did not feel confident in using DEALTS2 resources if they were not able to cover this content in the required depth. Moreover, this also had a detrimental impact on the quality of the training being delivered:

*‘If I had more time to deliver training, other elements from the toolkit would have been used’. (Interview 8)*

*‘Tricky to find time to fit it in. The time allowed for Tier 2 training is only 1.5 hours which is not enough time to effectively teach to a standard that I am happy with or that instils sufficient understanding in staff.’ (Interview 5)*.

Trainers described further difficulties that they had in getting staff released from duties on the ward to attend dementia training. Whilst trainers were passionate about delivering dementia training, it was evident that they felt deflated by the constant battle to fill course places with some trainers reporting half to two thirds of those registered attending on the day:

‘… *it is hard to get staff off the ward to train them’* (Interview 7).

The appetite for, and prioritisation of, dementia training was confounded by the fact that dementia is not a mandatory subject, despite the development of training frameworks published in national policy documents, such as DSTF [24, 25]. In some Trusts trainers described difficulties in implementing DEALTS2 because dementia was not a mandatory subject and was often deprioritised over training on mandatory subjects. One trainer suggested a way to overcome this would be to mandate dementia training.

#### Theme 4 - Trainer confidence and motivation

Trainers personal desire or need to apply the learning from the DEALTS2 TTT workshops to the dementia training being delivered in a Trust was a factor that appeared to support implementation. Overall, trainers liked the DEALTS2 toolkit and felt it is a good framework showcasing innovative dementia training approaches, lesson plans, styles of teaching, and evaluating the delivery and efficacy of the learning experience. Trainers that identified a need to start delivering higher level dementia training (Tier 2) in their employing Trusts and were keen to start using the toolkit and seemed more confident in adapting the simulation toolkit to suit their Trust training needs:

*‘I will use some elements. I am still delivering Tier 1 [general awareness]. It takes a long time to train everyone and need to complete this training first before Tier 2 [higher level] can be delivered’ (Interview participant 13).*

*‘It has been very useful. It is about picking out the elements that is relevant and incorporate them in the training that we deliver’* (Interview participant 8).

Conversely, trainers who appeared to lack this desire or need to apply the learning from DEALTS2 to the dementia training in their Trust were less likely to implement it. One trainer, for example, appeared to be satisfied with their existing training programme and so did not have a desire to use DEALTS2. Moreover, others appeared to lack the desire to introduce new materials into their current training package, or reflected a difficulty in knowing what content to add in an environment where resources are continually updated:

*‘I know a lot about dementia and have many resources available, which is constantly being updated’* (Interview 9).

Trainers with a desire or need to use the DEALTS2 simulation toolkit in their practice were more likely to feel confident in using the materials in their training when we followed up with them at least six months after the DEALTS TTT workshops.

### Impact on practice

Trainers who had implemented DEALTS2 in their practice were asked to described changes to their delivery of training as a result of their learning at the DEALTS2 workshops, as well as the impact on staff clinical practice, patient care and wider service improvement.

#### Theme 5 – Trainers enriched dementia teaching practice

Trainers described the impact of DEALTS2 TTT workshops and simulation toolkit on their teaching practice, related to increasing confidence in their own practice of teaching staff about dementia. This was connected to improvements in knowledge about dementia and exposure to new training approaches, which led some of the trainers to revise their dementia training to include the innovative new approaches they had been shown in the DEALTS2 TTT workshops:

*‘… I have been looking for new ideas, and the training came at the right time with a good range of activities.*’ (Interview participant 8).

Trainers liked the interactive aspects of the simulations. They valued the opportunity to participate in the simulations themselves, as this gave them a fresh perspective through first-hand experience, which enabled them to decide how they would implement DEALTS2 in their Trust dementia training:

*‘… it has made be think differently on how things can be delivered. It is always good to get new activities to use. (Interview participant 8).*

*‘It has given be more practical’s to use in my training sessions. These activities are very interactive which is good’* (Interview 1).

Trainers that had implemented DEALTS2 in practice described a positive impact on their teaching practice through increasing their confidence to teach staff about dementia through exposure to innovative interactive training approaches.

#### Theme 6 – Staff perceived to have enhanced dementia care approach

Some trainers acknowledged that it was difficult for them to assess the impact of DEALTS2 dementia training on staff clinical practice and wider service improvement. That said, trainers stated that staff were enjoying the training sessions and improving their knowledge of dementia:

*‘[Staff] have a deeper understanding now, they have more knowledge ‘(Interview 12)*.

There was a perception amongst some trainers that staff attending DEALTS2 dementia training had improved their clinical practice after attending training and that this meant they were supporting patients with dementia more effectively:

*‘…very good response [from staff] … they can apply to practice…. they have used the core of the training in their practice’ (Interview 12).*

Trainers valued how the simulation activities provided staff with an opportunity to participate in discussions about person-centred care. In addition, trainers felt that staff appreciated the opportunity to take part in the simulations and the debriefing afterwards, which enabled them to discuss and critique what they had learnt and link this back to their own practice in a safe environment away from the ward. It was this part of the training that the trainers really valued, and felt was a novel aspect that did not already happen within their existing training:

*‘The icebreaker activity was very good as the staff got more confident with each other…. the majority are very positive to the training and they are engaging….it was good to see how different individuals can relate to the scenarios and how they can apply this to [their own] practice’ (Interview 13)*.

Trainers felt the DEALTS2 training gave staff the opportunity to apply new knowledge and theory to their clinical practice, enabling them to better empathise with patients with dementia. This trainer’s suggested would lead to improved communication between staff and patients, as staff had increased understanding of dementia and insight into the patient experience following their attendance at training sessions. Trainers perceived that this would ‘plant a seed’ that would help staff to improve their practice and ultimately improve the care provided to patients with dementia:

‘. *the training has made [staff] more aware and they think twice about it, they have a deeper understanding…. they seem better in how to communicate with patients. It has led to better communication between patients and staff*.’ *(Interview participant 9).*

Trainers reported that staff were more engaged following training, improving their empathy with patients and the care delivered. One trainer also noted a decrease in waiting times for patients with dementia:

*‘… staff commitment seems better; they are more engaged after training… [and there is a] quicker flow with patients in waiting room’* (Interview participant 11).

## Discussion

To our knowledge, this study is the first theory and evidence-based dementia training intervention using an innovative simulation-based toolkit approach and delivered through a TTT model, to be evaluated nationally across England, capturing the perspectives of trainers implementing the intervention. The aim of this paper was to explore trainer perspectives of the barriers and enablers experienced when implementing the DEALTS2 dementia training intervention across England, and the impact on delivery of training and staff clinical practice following implementation. Our findings show that trainers who felt supported by their line managers and senior leadership teams with adequate time, resources (such as equipment, physical space and colleagues to help with delivery), and personal responsibility to deliver effective dementia training were more likely to feel confident in implementing DEALTS2 in practice. These factors were identified by many of our participants, who were geographically spread across England (with representation from most HEE regions) and included those who were more and less experienced in terms of delivering dementia training. The national scale of our study and the inclusion of acute hospitals with less experience in terms of dementia training delivery, adds further weight to the findings of a previous study which identified components of impactful dementia training for hospital staff (based on case studies from three NHS Trusts in England which were high ranking in terms of dementia training delivery) [7].

The aim of DEALTS2 was to utilise simulation and experiential learning approaches to enable trainers to put staff into the shoes of a person with dementia, therefore understanding more about the lived experience. Most trainers were positive about the simulations and the value of this approach to gain insight into the lived experience of dementia and develop interpersonal skills, as has been found in previous smaller studies [15, 17]. Many trainers felt the flexible toolkit approach was key to them using the DEALTS2 materials in the training they were delivering to staff in their trust, as they were able to adopt, or adapt, the simulations to suit local training needs in their acute hospital. A small number appeared less confident in being able to adapt them to suit the needs of different staff groups. Our findings demonstrate the need for simulations to be adaptable to suit different staff groups and for trainers to feel confident in assessing appropriateness within the time available to them, which again adds further evidence to support the findings of the case study review based on three NHS Trusts in England [7]. Trainers described the debriefing after the simulation as a key aspect in the success of the training that enabled staff an opportunity to discuss and critique what they had learnt and connect this to their own practice in a safe environment away from the ward. This builds on the ASPiH and HEE [38] guidance that identifies the need for structured debriefing sessions after using simulations in training, highlighting the real-life benefit of this for staff.

Furthermore, trainers suggested that in the future they would value additional training materials to support the delivery of DSFT [25, 26] in subject areas not covered by the DEALTS2 simulation toolkit. Given the need to ensure that DEALTS2 training did not cover too many learning outcomes, as programmes with fewer outcomes are reported by staff as more beneficial, the programme focused on three Tier 2 subject areas [32]. In accordance with other research identifying a lack of training covering the subjects ‘pharmacological interventions in dementia care’, ‘leadership’ and ‘end of life care’, we suggest these to be the ideal starting point for anyone developing further tier 2 dementia training resources in the future [6]. Whilst this training was delivered face-to-face there maybe a need to look at other delivery approaches (such as e-learning and online delivery) to support blended learning delivery of the plethora of subjects outlined in the DSFT [25, 26]. Albeit including simulation and debriefing, given how much trainers felt that staff valued these aspects of DEALTS2.

### Implications for policy and practice

Undervaluing the amount of time that it takes it deliver effective dementia training beyond general awareness level, combined with a lack of management and organisational support, was described by some trainers as a barrier to implementing DEALTS2. This is potentially detrimental to the overall quality of any dementia training being delivered. Our study findings highlight gaps nationally in terms of the prioritisation and consistency of delivery of dementia training across the acute care workforce in England. We have shown a potential disconnect between dementia training policy, as outlined in national frameworks such as DSTF [25, 26], and current practice in some Trusts where trainers described a lack of adequate time to cover higher level dementia training content in the required depth. For example, some reported needing to cover all subjects at Tier 2 of the DSTF [25, 26] in a timeframe of only one and a half hours. Not providing effective dementia training at any level has implications for practice, including a negative effect on the quality of care provided to patients, as well as staff well-being and overall job satisfaction [32, 39]. Our findings give a novel insight into the dementia training currently being delivered in some acute hospitals in England. This context is insightful given that previously time has been described much more abstractly as a barrier to implementing dementia training, without evidence of the actual periods some trainers have available to them to deliver such training [20].

Dementia training is currently not a mandatory requirement and therefore it can, and is, deprioritised over other mandatory subjects in some acute hospitals. To ensure high-quality and consistent delivery of dementia training across acute care nationally in the long-term, there is a need for mandatory accredited dementia training [6]. In the short-term though we suggest a need to raise awareness of key United Kingdom (UK) dementia training policy guidance documents and training frameworks [25, 26, 40] at all levels across acute hospitals. Moreover, it would be beneficial to signpost examples of peer reviewed dementia training toolkits and materials (such as DEALTS2) within these policy documents and training frameworks, providing additional support and guidance to individuals in training roles to enable them to proactively develop and enhance their teaching practice [2, 32].

### Strengths and limitations

This study draws upon the experiences of trainers delivering innovative simulation-based dementia training in acute care settings nationally across England. The qualitative approach applied gives voice to trainers, enabling them to share their experiences of the barriers and enablers faced when implementing DEALTS2 informed dementia training in their employing Trust, and the resulting impact on training delivery and staff clinical practice. We have focused on trainers’ perspectives of implementing and delivering a TTT dementia training intervention within acute hospitals, unlike previous studies that have reported staff perspectives of receiving such interventions [41–44]. As such, our findings contribute new perspectives to wider debates about the effectiveness of dementia training in acute care settings [2, 6, 7, 20, 18, 32].

Whilst our findings may not be representative of all trainers that took part in the DEALTS2 TTT workshops, we were able to hear the experiences of 17 trainers with a good spread of representation nationally across HEE regions, and did reach saturation point. The interviews ranged from 15 to 32 min, excluding consent processes, and we acknowledge that these were relatively short for a qualitative study. By taking this approach we were able to reach some of the trainers to seek their views, ensuring that they were able to find the time to share their experiences. However, others declined taking part in interviews for the following reasons: working in a new job role not focusing on training delivery, lack of time or forthcoming annual leave, and not working on the same days as the part-time researcher. We used self-reported measures of perceived impact on training delivery and staff clinical practice and were successful in terms of reaching our participants who were able to clearly identify how their learning had enhanced their practice. We note that when reporting on the impact on staff clinical practice, not all trainers may have had contact with the staff they had trained after the training, and so may of been reporting staff intentions rather than actions. We recognise though that there is a need for further research on behaviour change to determine care improvements more specifically [32].

## Conclusions

The focus of this paper on trainer experiences to implement dementia training in acute care settings provides novel perspectives, highlighting the individual and organisational barriers and enablers faced by trainers when implementing the DEALTS2 dementia training intervention across England. Our findings stress the importance of support from line managers, senior leadership teams and organisational culture to ensure effective and impactful dementia training. One key aspect that enabled implementation of DEALTS2 was the flexible toolkit approach, which supported adherence amongst trainers by giving them the ability to adopt, or adapt, the materials to suit local training needs in their acute hospital. Further implementation enablers were: the simulation approach and debriefing afterwards that put staff into the shoes of a person with dementia and enabled them to discuss and critique what they had learnt and link this back to their own practice in a safe environment away from the ward; use of low-key simulations that did not rely on technology or other resources to implement; organisational level factors like trainers having enough time with staff to deliver effective training; and trainers individual confidence and motivation. To ensure wider implementation of DEALTS2 nationally there is a need to raise awareness and understanding of policy guidance documents and training frameworks at all levels of management and amongst practitioner’s and staff within acute hospitals across England; and of the time it takes to deliver effective and impactful dementia training, particularly beyond general awareness level. Further research should explore staff perspectives of DEALTS2 dementia training, measure behaviour change and the impact of such training on patients with dementia and their families.

For more information about the study please visit the project website at: https://www.bournemouth.ac.uk/research/projects/dementia-education-learning-through-simulation-2-dealts-2-programme.

## Data Availability

The data that support the findings of this study are available from Bournemouth University, but restrictions apply to the availability of these data, which were used for the current study, and so are not publicly available. Data are however available from the corresponding author upon reasonable request and with permission of Health Education England.

## Abbreviations

DEALTS2: Dementia Education And Learning Through Simulation 2
DSTF: Dementia Standards Training Framework
DeTDAT: Dementia Training Design and Delivery Audit Tool
HEE: Health Education England
HVF: Humanisation Values Framework
NICE: National Institute for Health and Care Excellence
NHS: National Health Service
TTT: Train-The-Trainer
UK: United Kingdom
